# Evolving relationship of Nares Strait ice arches on sea ice along the Strait and the North Water, the Arctic’s most productive polynya

**DOI:** 10.1038/s41598-023-36179-0

**Published:** 2023-06-17

**Authors:** G. W. K. Moore, S. E. L. Howell, M. Brady

**Affiliations:** 1 Department of Physics, University of Toronto, Toronto, Canada; 2 Department of Chemical and Physical Sciences, University of Toronto Mississauga, Mississauga, Canada; 3 Climate Research Division, Environment and Climate Change Canada, Toronto, Canada

**Keywords:** Cryospheric science, Climate-change impacts

## Abstract

Nares Strait, the waterway that separates northwest Greenland from Ellesmere Island, is a major pathway along which sea ice leaves the Arctic, including the planet’s oldest and thickest sea ice that is experiencing an accelerated loss. Ice arches that develop during the winter at the Strait’s northern or southern terminus can remain stable for months at a time during which the transport of sea ice ceases. The Arctic’s most productive polynya, the North Water (NOW) or *Pikialasorsuaq* (West Greenlandic for ‘great upwelling’) forms at the Strait’s southern end. There is evidence that a warming climate and the concomitant thinning of Arctic sea ice is weakening the arches and it has been proposed that this may impact the stability of NOW and the complex ecosystem that it sustains. Here we employ a categorization of recent winters with respect to the presence or absence of ice arches to explore their impact on sea ice along the Strait and over the NOW. We find that winters during which a southern ice arch is absent are associated with a reduced and thinner ice cover along the Strait with ice conditions over the NOW similar to that during winters with a southern arch. In winters, without a southern arch, there is also an acceleration of the winds along the Strait that contributes to the presence of reduced ice cover. Ocean color remote sensing data suggests that current levels of primary productivity over the NOW are independent of the presence or absence of an ice arch. The results suggest more research is needed to assess the stability of the NOW, with respect to reduced ice cover and primary productivity, in a future where ice arches cease to form along Nares Strait.

## Introduction

**Figure 1:**
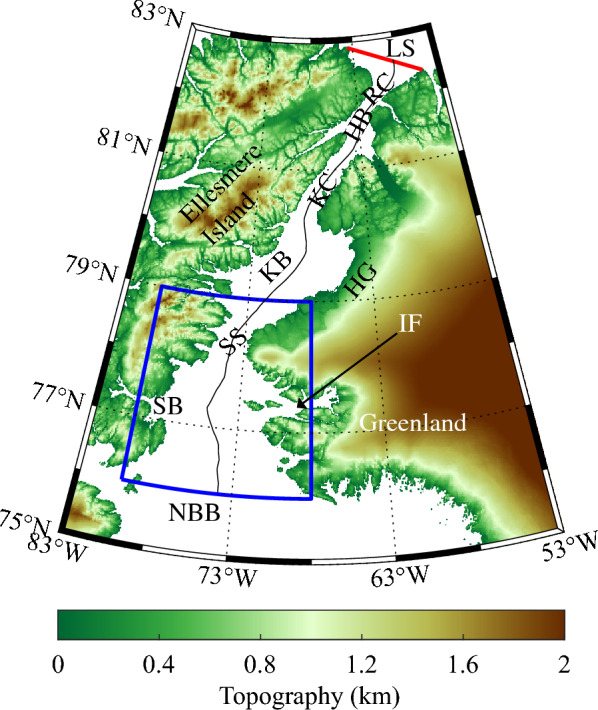
Topography (km) of the Nares Strait region as represented in the GEBCO Digital Elevation Model. The abbreviations for locations along the strait are: northern Baffin Bay (NBB); Smith Bay (SB); Inglefield Fjord (IF); Smith Sound (SS); Kane Basin (KB); Humboldt Glacier (HG); Kennedy Channel (KC); Hall Basin (HB): Robeson Channel (RC) and the Lincoln Sea (LS). The thick red line indicates the location of the flux gate used to characterize ice motion along Nares Strait. The blue polygon represents the approximate location of the North Water. The thin black curve represents the centerline of Nares Strait.

Nares Strait is the waterway that separates northwest Greenland from Ellesmere Island (Fig. 1). Along with Fram Strait, to the east of Greenland, and the Canadian Arctic Archipelago, to the west of Ellesmere Island, it is a major pathway for the export of Arctic sea ice^1,2^ and freshwater^3^ southwards into sub-arctic waters impacting ocean salinity and dynamics^4^. The planet’s oldest and thickest sea ice is situated to the north of Nares Strait in a region known as the Last Ice Area that is predicted to be the last region in the Arctic to lose its perennial ice cover^5,6^. The export of this ice, that has seen an accelerated loss in recent years^7^, occurs along the Strait^1,8^.

The narrowness of the Strait when combined with the mechanical properties of sea ice, most notably its high uniaxial compressive strength, results in the frequent formation of arch-like features that can remain stable for months at a time^9,10^. Typically, the arches form in December or January and remain stable until July or August with their presence eliminating the export of sea ice along Nares Strait^1,8^. Arches can form either at the northern end of the Strait, to the north of Robeson Channel^10^, or in the vicinity of Smith Sound at its southern end^1^. Southern arches are more common during the satellite record^11^. The uniaxial compressive strength of sea ice is a function of its thickness^9^ and so there is concern that observed thinning of Arctic sea ice in the region to the north of Nares Strait may be impacting the arch’s stability^12–14^.

The North Water (NOW) or *Pikialasorsuaq* is a recurring polynya situated just south of Smith Sound (Fig. 1). It has been argued that the presence of a southern ice arch, by limiting the southward transport of sea ice, is required for the existence of the NOW^14–17^. High winds that arise due to flow distortion associated with the narrowness of Smith Sound also contribute to the presence of open water by sweeping any sea ice downstream^16,18,19^. In addition, the wind-driven upwelling of warm Atlantic water, in combination with convective entrainment triggered by brine rejection, also contributes to its formation^20,21^.

The NOW is the Arctic’s largest and most productive polynya^14,22^. The reduction in ice cover along with the nutrients provided by upwelling contribute to its productivity^20,23^. The ice free waters support early blooms of phytoplankton that are the basis of a productive ecosystem that supports important species that include: Arctic cod, seabirds, as well as seals, walruses, narwals, beluga and polar bears^14,23,24^. There is evidence that the NOW is expanding in size as a result of warming air and sea surface temperatures in the region^25,26^. In addition, ocean color remote sensing of chlorophyll-a (Chl-a) indicate that phytoplankton blooms over the NOW typically start in late April and early May, reach peak amplitudes in late May and June before declining through July to September^27,28^. In addition, the bloom duration is positively correlated with the period of open water^27^. During the winter of 2009, a northern ice arch formed and this lead to low ice cover over the NOW and a particularly early bloom^27^. The amplitude of the bloom, defined as the peak value of Chl-a, has a negative trend that may be related to changes in sea surface conditions, increases in sea surface temperatures and decreases in sea surface salinity, as well as changes in wind driven upwelling of nutrients^27,28^. There is also concern that the weakening of the ice arches along Nares Strait may impact the stability of the NOW, its high level of productivity and its role as a traditional food source for nearby Inuit communities^1,14,24^. In this regard, a modelling study^29^ suggests that changes to the ocean, including an increase in sea surface temperatures through the upwelling of Atlantic Water, a shallowing of the mixed layer and an increase in stratification as well as an increase in ice flux, will fundamentally alter the nature of the NOW with potential impacts on its productivity. Indeed, the warming of Atlantic Water has been argued to contribute to a recent delay in ice growth in the region during the fall^30^.

Satellite-based synthetic aperture radar (SAR) imagery together with feature tracking algorithms^31^ provide a way to document the ice transport along the Strait^1,8^. With this technique it was noted that no arches formed along Nares Strait during the winter of 2007^1^. This absence allowed for ice transport throughout the winter and as a consequence annual ice area and volume fluxes were twice as large as the mean over 1997–2009^1^. Using SAR imagery, 2019 was also identified as a winter in which ice transport continued throughout the winter and as a consequence, no ice arches formed along the Strait^8^.

Variability of sea ice along Nares Strait has not been extensively studied. However there is significant body of historical knowledge regarding this variability that is related to expeditions during the nineteenth century that attempted to reach the North Pole via the Nares Strait^32^. In June 1854 during the Second Grinnell Expedition, Kane observed the presence of open water in Kennedy Channel that he that he assumed was the start of the Open Polar Sea^33^. Subsequent expeditions such as Hall’s Polaris Expedition of 1871–1873 and Nares’ British Arctic Expedition of 1875–1876 were able to navigate through sea ice all along Nares Strait up to the Lincoln Sea where thick ice blocked further progress^34,35^. Bessels, the Chief Scientist on the Polaris Expedition, noted that Nares Strait was never entirely frozen over including Hall Basin where open water and wind-driven mobile ice were observed during their overwintering^34^.

More recently, a polynya was observed to form to the south of a northern ice arch during the winter of 1989^10^. A similar polynya was observed to develop to the south of a northern ice arch during the winter of 2017^36^. Examples using daily infra-red satellite imagery^37^ indicate that during May there was open water along Nares Strait during a winter when no southern ice arch formed, i.e. either a northern ice arch or no ice arch, that was in contrast to the extensive ice cover along the Strait during a winter in which a southern ice arch formed. This result was extended in a study^8^ that showed monthly mean sea ice concentration maps for June 2018 and 2019. During the winter of 2018, a southern ice arch formed; while during the winter of 2019 no arches formed. During June 2018, Nares Strait had close to 100% ice cover north of Smith Sound. In contrast during June 2019, the Strait had open water as far north as 81° N.

It has been shown that southern arches along Nares Strait tend to form during periods of cold surface air temperatures around low tide and during a cessation or even a reversal of the prevailing southerly winds along the Strait^38^. In addition, ICESat-2 freeboard data has been used to show that sea ice was thinner during the winter of 2019, when no southern arch formed, as compared to the winter of 2020, when a southern arch formed^38^. Warming subsurface waters of Atlantic and Pacific origin have been argued to contribute to a reduction in thermodynamic ice growth throughout the winter along Nares Strait thereby contributing to the observed weakening of arches along Nares Strait^39^.

As we will show in this paper, the winter of 2022 was another year in which ice motion continued throughout the winter along Nares Strait and as a result, no arches formed. We will use recent winters in which: a southern arch formed, a northern arch formed or no arches formed to assess the impact that ice arches have on the distribution of sea ice along Nares Strait and the NOW. We find that the absence of a southern ice arch results in the presence of open water and thin ice along Kennedy Channel. While over the NOW in the absence of a southern ice arch, there is a similar distribution of sea ice as well as similar levels of primary productivity as occurs when a southern ice arch is present.

## Results

### Updated Nares Strait ice area flux

**Figure 2:**
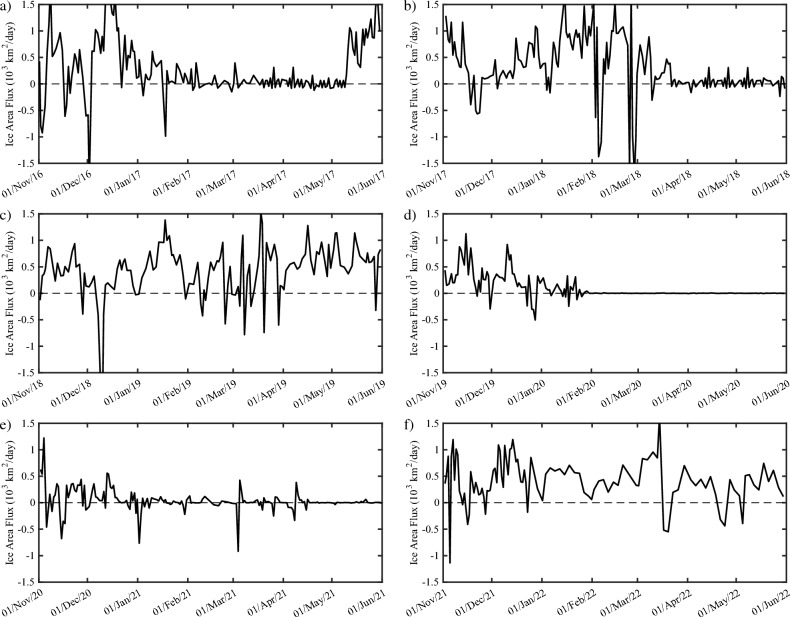
Time series of the ice area flux (10^3^ km^2^/day) across the Lincoln Sea flux gate. Results are shown for the winters of: (**a**) 2017; (**b**) 2018; (**c**)2019; (**d**) 2020; (**e**) 2021 and (**f**) 2022. Positive/negative fluxes represent southwards/northwards ice motion. The change in the frequency of sampling that occurred in Jan 2022 was the result of the loss of data from Sentinel-1B.

Figure 2 provides an updated time series of the winter (defined as November 01–June 01) ice area flux (IAF), please refer to the Methods Section for information on the calculation of the IAF, across the southern Lincoln Sea flux gate (Fig. 1) that extends previous results^8^ to include the winters of 2020, 2021 and 2022. The presence of an ice arch is indicated by a marked reduction in the IAF^1,8^. In addition to the previously identified cessation of this flux during 2017 and 2018^8^, there was also a cessation during 2020 and 2021. There was variability in the nature of the IAF during these 4 winters that ranges from a complete cessation of the IAF during the winter of 2020 to continued small magnitude fluctuations during the remaining 3 winters that speaks to an underlying variability in the stability of the arches that will be the subject of a future investigation. As was the case during 2019, the IAF remained large during the winter of 2022 indicating the absence of an arch along the Strait.

**Figure 3:**
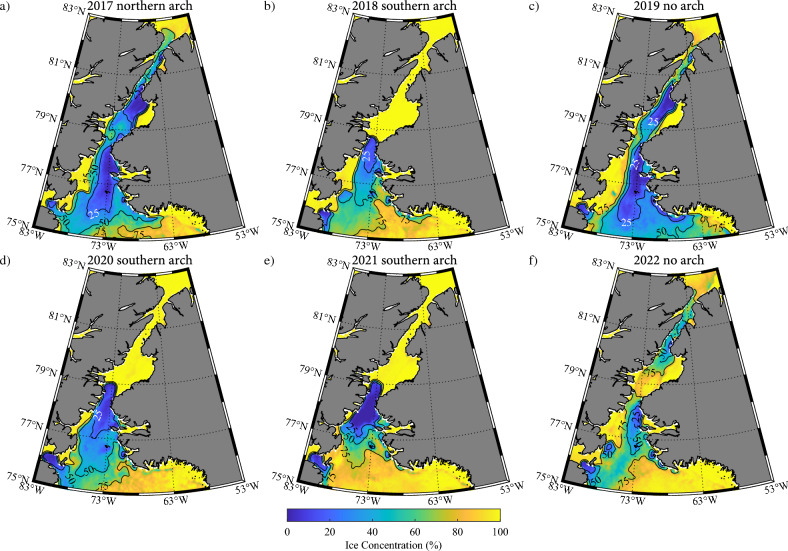
The monthly mean sea ice concentration (%) during May: (**a**) 2017; (**b**) 2018; (**c**) 2019; (**d**) 2020; (**e**) 2021 and (**f**) 2022. The corresponding winters are categorized as to the presence or absence of an ice arch.

In Fig. 3, we present the monthly mean sea ice concentration, please refer to the “Methods” section for information on the sea ice concentration dataset, during May for the years during which the IAF was shown in Fig. 2. May was chosen as it represents one of the last months during the winter during which arches are typically still present^8,37^ as well the first month of the year in which there is significant primary production in the NOW^27^. It is evident that the sea ice concentration along Nares Strait north of Smith Sound is close to 100% with reduced ice cover over the NOW during winters with a southern ice arch (Fig. 3b,d,e). In contrast, winters with a northern ice arch present (Fig. 3a) or no ice arches present (Fig. 3c,f) have reduced ice cover along the Strait, in particular along the Kennedy Channel, as well as over the NOW.

### Impact of the ice arches on sea ice concentration along Nares Strait and the NOW

**Figure 4:**
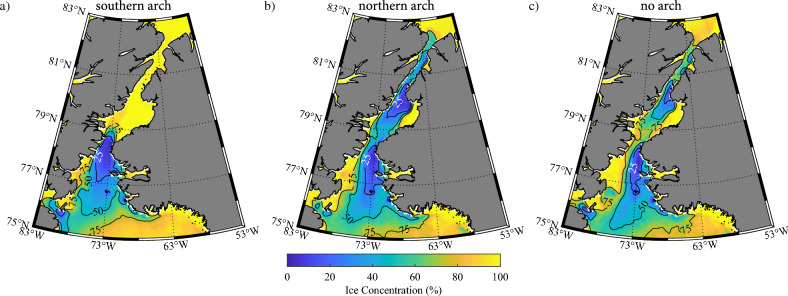
Climatological sea ice concentration along Nares Strait during May. Results are shown for: (**a**) winters in which a southern ice arch formed; (**b**) winters in which a northern ice arch formed and (**c**) winters in which no ice arch formed. Please refer to the “Methods” section for details on the categorization of the winters. All fields from the AMSR-E/2 Artist dataset that is available from 2002 to 2022.

Figure 3 provides evidence as to the impact that various ice arch categories have on the distribution of sea ice along the Strait as well as over the NOW. There are differences between the winters in the various categories that are the result of inter-annual variability. To reduce the impact of this inter-annual variability as well as to quantify the impact that the various arch permutations have on the distribution of sea ice along Nares Strait and over the NOW, composites were created for: winters with a southern arch; winters with a northern arch and winters without an arch. Please see the Methods Section for details on the categorization of the winters. Figure 4 shows composite monthly mean sea ice concentrations during May for these three categories.

The presence of a southern arch (Fig. 4a) is characterized by close to 100% ice cover to the north of Smith Sound with reduced ice cover associated with the NOW as far south as ~ 76° N. Within northern Baffin Bay, there is close to 100% ice cover along the Ellesmere Island coast in the vicinity of Smith Bay as well as in the vicinity of Inglefield Fjord along the Greenland coast (Fig. 1). The location of the arch can vary from year-to-year^37^ and as a result, its location is represented by the diffuse transition from 0 and 100% ice cover to the north of Smith Sound. In contrast, the presence of a northern arch (Fig. 4b) results in concentrations as low as 25% along much of the Strait except for eastern Kane Basin where ice cover remains close to 100%. To the south of Smith Sound, there is an expansion of the region of close to 100% ice cover in the vicinity of Smith Bay (Fig. 1). However, there is still reduced ice cover over the NOW. The situation for the case when there were no arches present (Fig. 4c) is similar to that for the northern arch category (Fig. 4b) with the exception that there is more ice cover along Robeson Channel, southern Kane Basin and Smith Bay.

**Figure 5:**
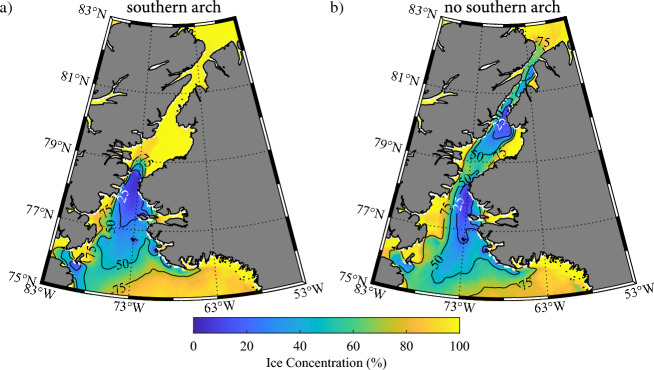
Climatological sea ice concentration along Nares Strait during May. Results are shown for: (**a**) winters in which a southern ice arch formed; (**b**) winters in which a northern ice arch formed and (**c**) winters in which no ice arch formed. Please refer to the “Methods” section for details on the categorization of the winters. All fields from the AMSR-E/2 Artist dataset that is available from 2002 to 2022.

Given the similarity in ice cover between the case where a northern ice arch is present and no arch is present, we will collapse these two categories into a single category characterized by the absence of a southern arch. Figure 5 presents the composites for the southern arch and no southern arch categories. The similarities in the sea ice concentration south of Smith Sound over the NOW between the two categories is evident with ice concentrations less than 25% on the eastern side of northern Baffin Bay. In contrast, the sea ice concentration north of Smith Sound is in excess of 75% for the southern arch categories while it is again less than 25% in the northern Kane Basin and southern Kennedy Channel.

**Figure 6:**
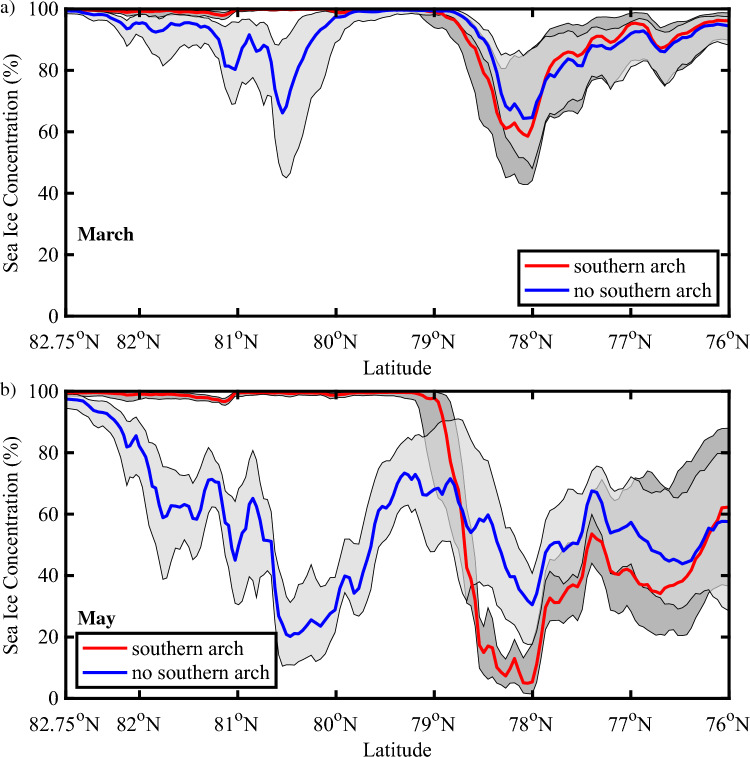
Spatial variability in sea ice concentration (%) along Nares Strait. Results are shown for: (**a**) March and (**b**) May. The red lines indicate the median ice concentration for years in which a southern arch formed with the dark shading representing the ice concentrations bounded by the first and third quartiles. The blue lines indicate the median ice concentration for years in which no southern arch formed with the light shading representing the ice concentrations bounded by the first and third quartiles. Please refer to the “Methods” section for details on the categorization of the winters. All fields from the AMSR-E/2 Artist dataset that is available from 2002 to 2022.

To quantify the differences in ice cover along the Strait and over northern Baffin Bay between the two categories, we present in Fig. 6 the average ice concentration along the Strait and over the NOW as a function of latitude during both March and May. Please refer to the “Methods” section for information on the methods used in the latitudinal averaging. A measure of the year-to-year variability for each category is provided.

During March (Fig. 6a) for years in which a southern arch is present, ice cover is close to 100% with only a small amount of variability from year-to-year north of ~ 79° N, the approximate location of the southern ice arch (Fig. 5a). Sea ice concentration reaches a minimum of ~ 60% in the vicinity of ~ 78° N before rebounding to close to 100% by 76° N. This region of reduced ice cover represents the NOW. The situation during March for years with no southern arch present is different (Fig. 6a) with reduced ice cover north of ~ 79° N that reaches a minimum of ~ 60% in the vicinity of the Kennedy Channel, ~ 81° N, (Fig. 1). Over the NOW, i.e. south of ~ 79° N, the distribution of sea ice is similar to that in the southern arch category.

The situation during May (Fig. 6b) is qualitatively like that in March with sea ice concentrations lower, less than 25%, for the no southern arch category north of ~ 79° N. In contrast, ice cover for the southern arch category remains close to 100% in this region. In the southern arch case, there is a steep drop in ice concentrations, from ~ 100 to < 20%, south of 79° N. This represents the presence of the arch. For the no arch case, there is a more moderate decrease in ice concentrations, from ~ 60 to ~ 40%, in this region. South of 78° N, ice concentrations tend to rebound to higher values with ice concentrations in the no arch case consistently higher by ~ 10–20%.

**Figure 7:**
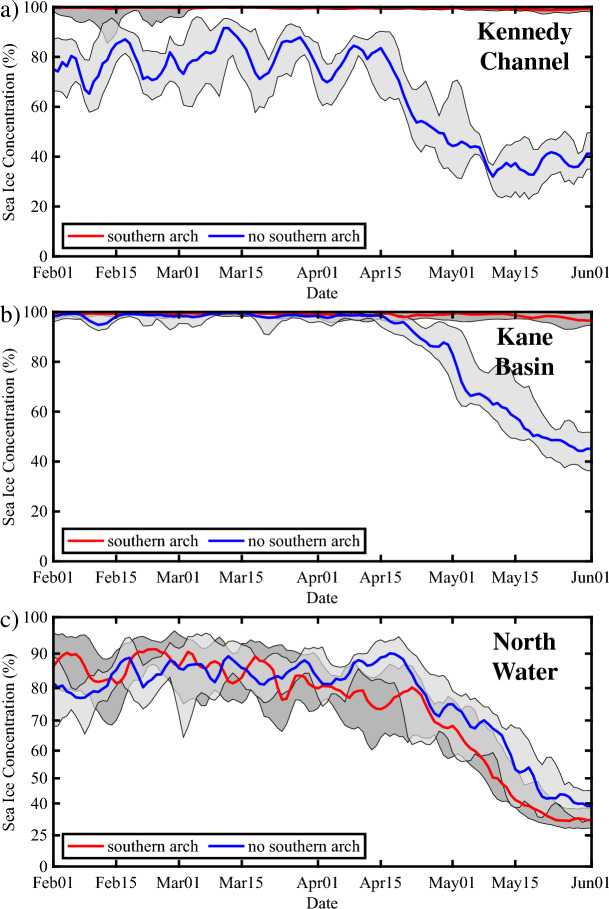
Time series of sea ice concentration (%) along sections of Nares Strait during the period Feb 01 to Jun 01. Results are shown for the: (**a**) Kennedy Channel 80°–81°N; (**b**) Kane Basin 79°–80° N and (**c**) North Water 77°–79° N. The red lines indicate the median ice concentration for years in which a southern arch formed with the dark shading representing the ice concentrations by the first and third quartiles. The blue lines indicate the median ice concentration for years in which no southern arch formed with the light shading representing the ice concentrations bounded by the first and third quartiles. Please refer to the “Methods” section for details on the categorization of the winters. All fields from the AMSR-E/2 Artist dataset that is available from 2002 to 2022.

To provide more details on the seasonal evolution of the ice cover, we present in Fig. 7, time series for the period February 01 to June 01 of the ice cover and its year-to-year variability for both categories averaged along Kennedy Channel (80°–81° N), as well as along Kane Basin (79°–80° N) and over the NOW (76°–79° N). For the Kennedy Channel (Fig. 7a), ice cover during the southern arch category remains close to 100% throughout this period with little year-to-year variability. In contrast, ice cover in this region for the no southern arch category is on the order of 80% up to mid April after which it undergoes a reduction reaching ~ 40% by June 01. To the south of Kennedy Channel over Kane Basin (Fig. 7b), ice cover in both categories remains close to 100% up until mid April, after which it reduces to ~ 50% by June 01. Over the NOW (Fig. 7c), ice cover for both categories is on the order of 80% up to mid April. After this time, ice cover is reduced for both categories. Consistent with the results from Fig. 5, ice cover during this period is consistently higher for the no southern arch category by ~ 10–20%.

**Figure 8:**
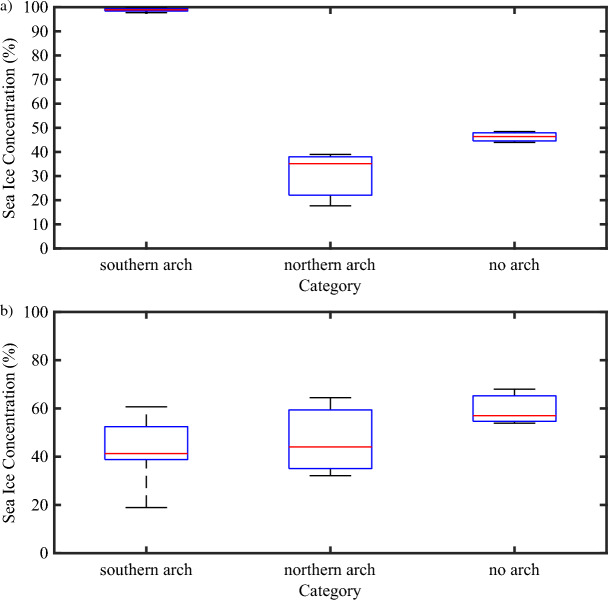
Box and whisker plot of the monthly mean sea ice concentration (%) along sections of Nares Strait during May. Results are shown for the: (**a**) Kennedy Channel 80°–81° N; and (**b**) North Water 76°–79° N. All fields from the AMSR-E/2 Artist dataset that is available from 2002 to 2022.

There is a concern that the averaging over years in the no southern arch category may mask differences between the no arch and northern arch categories. To mitigate these concerns, we present in Fig. 8, box and whisker plots of the monthly mean sea ice concentration during May for the Kennedy Channel (80°–81° N) and the NOW (77°–79° N). With respect to the Kennedy Channel (Fig. 8a), it is clear that all the years with either a northern arch or no arch are outliers, characterized by sea ice concentrations varying from 10 to 50%, with respect to the southern arch years, characterized by sea ice concentrations close to 100%. Ice concentration along the Kennedy Channel are lowest for the northern arch years as compared to the no arch years. This is consistent with the composites shown in Fig. 5. The situation along the NOW (Fig. 8b) is more nuanced with sea ice concentrations for the southern or northern arch years generally lower than those for the no arch years. There is however considerable inter-annual variability that can result in similar ice concentrations amongst the three categories.

### Impact of the ice arches on sea ice thickness along Nares Strait and the NOW

**Figure 9:**
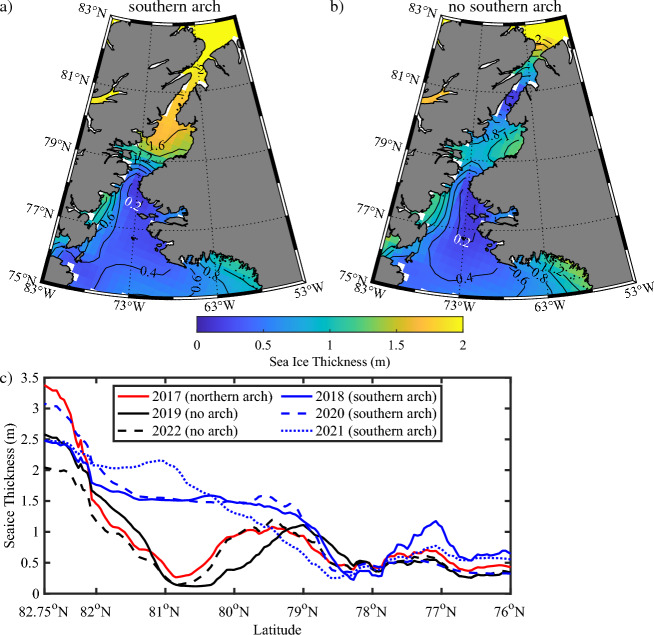
Impact of arches on the sea ice thickness along Nares Strait. Climatological sea ice thickness during during April for: (**a**) years in which a southern ice arch formed and (**b**) no southern ice arch formed. (**c**) Spatial variability in sea ice thickness along Nares Strait during April for years with a northern arch, 2017, no arch present, 2019 and 2022, and a southern arch present, 2018, 2020 and 2021. All data from the AWI Cryosat2-SMOS dataset 2011–2022.

Following the approach used above, the sea ice thickness along Nares Strait were composited over winters with and without a southern arch (Fig. 9). Please see the “Methods” section for details on the ice thickness dataset used. The shortness of the sea ice thickness dataset, 2011–2022, does not allow for as robust analysis as was the case for sea ice concentration. The focus will be on the sea ice thickness during April as this is the last month during the winter for which data is available^40^. The composite for winters with a southern arch present (Fig. 9a), indicates that there is a transition in thickness from close to 0 m over the NOW to ~ 1.25 m over Kane Basin. North of Kane Basin, the thickness increases obtaining values in excess of 2 m over the Lincoln Sea. Along the western boundary of the NOW, the sea ice thickness is ~ 0.5 m. The composite for winters without a southern arch (Fig. 9b), in contrast, indicates that the thicknesses along Kane Basin are less than 1 m while those along Kennedy Channel are less than 0.5 m. North of Kennedy Channel, the thicknesses increase to values in excess of 2 m over the Lincoln Sea. As was the case for the southern arch case, thicknesses along the western boundary of the NOW were ~ 0.5 m.

The spatial variability during April along Nares Strait and the NOW for recent years (Fig. 9c) confirms the separation in ice thickness for years with and without a southern arch. This is especially true along the Kennedy Channel, ~ 81° N, where thicknesses during years with a southern arch are in excess of 2 m; while they are less than 0.5 m during years without an arch. South of Kennedy Channel in the vicinity of Kane Basin, ~ 81°–79° N, the thickness for the no southern arch case increases to values commensurate with that for the southern arch case.

### Impact of the ice arches on winds along Nares Strait and the NOW

**Figure 10:**
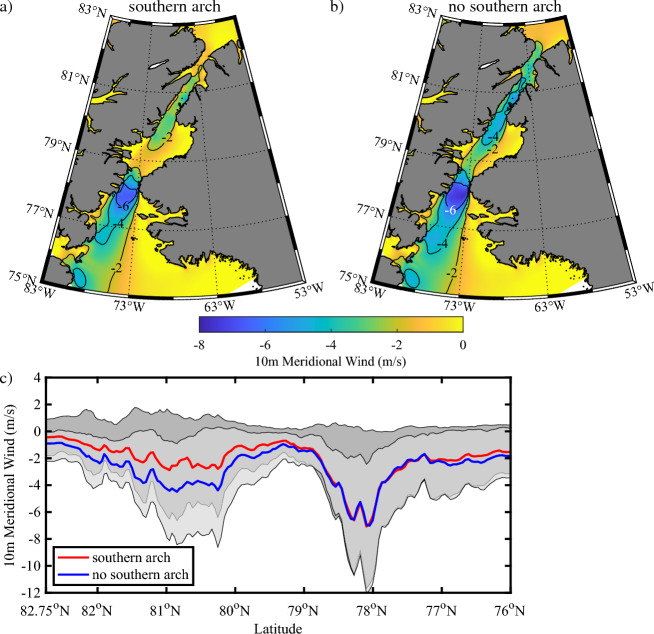
Impact of arches on the 10 m winds along Nares Strait. Climatological 10 m meridional wind during during May for: (**a**) years in which a southern ice arch formed and (**b**) no southern ice arch formed. (**c**) Spatial variability in 10 m meridional wind along Nares Strait during May. The red lines indicate the median 10 m meridional wind for years in which a southern arch formed with the dark shading representing the 10 m meridional winds bounded by the first and third quartiles. The blue lines indicate the median 10 m meridional winds for years in which no southern arch formed with the light shading representing the 10 meridional winds bounded by the first and third quartile. Please refer to the “Methods” section for details on the categorization of the winters. All data from the CARRA 2002–2022.

The results presented above indicate that there are two regions along Nares Strait where low sea ice concentrations and thin ice are possible: to the south of Smith Sound as well as along the Kennedy Channel. It is well known that flow distortion associated with the narrowness of Smith Sound leads to an acceleration of the local wind^19,41^. It has also been shown that Kennedy Channel is another region along Nares Strait where wind speeds are elevated^42,43^. Given these colocations, it is likely that these localized regions of high winds contribute to these minima in sea ice concentration and thickness. This is confirmed in Fig. 10 which shows the impact that the presence or absence of a southern ice arch has on the meridional component of the wind along the Strait and over the NOW during May. Please refer to the “Methods” section for details on the dataset used to produce this figure. The southern arch category (Fig. 10a) indicates that Smith Sound and the NOW are locations where the northerly winds are highest along the Strait. A secondary maximum is present along Kennedy Channel and northern Kane Basin. The situation is similar for the no southern arch category (Fig. 10b) with the exception that the magnitude of meridional wind along Kennedy Channel and northern Kane Basin are higher by ~ 2 m/s.

The distribution of the 10 m meridional winds along the Strait and over the NOW for both categories (Fig. 10c) confirm that the meridional winds along the Kennedy Channel are elevated when a southern arch is absent. The significance of this difference was assessed by comparing the magnitude of the meridional wind for the no arch category against the distribution for all combinations of 6 years selected from the set of all years with a southern arch. Along the Kennedy Channel, it was found that the difference was statistically significant (p < 0.02).

### Impact of the ice arches on primary productivity along Nares Strait and the NOW

**Figure 11:**
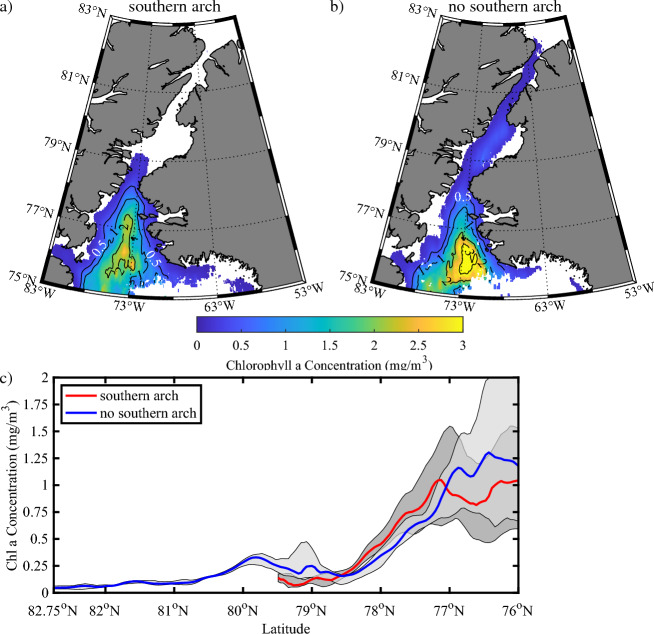
Impact of arches on primary productivity along Nares Strait. Climatological Chl-a concentration during during May for: (**a**) years in which a southern ice arch formed and (**b**) no southern ice arch formed. (**c**) Spatial variability in Chl-a concentration along Nares Strait during May. The red lines indicate the median Chl-a concentration for years in which a southern arch formed with the dark shading representing the Chl-a concentration bounded by the first and third quartiles. The blue lines indicate the median Chl-a concentration for years in which no southern arch formed with the light shading representing the Chl-a concentration bounded by the first and third quartiles. Please refer to the “Methods” section for details on the categorization of the winters. All data based on the Aqua/Terra/Suomi NPP Chl-a data 2002–2022.

In Fig. 11, we present the satellite retrievals of Chl-a, please refer to the “Methods” section for information on this dataset including its limitations, during May for the southern arch and no southern arch categories. When a southern arch is present (Fig. 11a), the presence of Chl-a is restricted to the ice free regions of the NOW (Fig. 4a) with values in excess of 1.5 mg/m^3^ occurring in the central northern Baffin Bay. In the absence of a southern arch (Fig. 11b), the distribution of Chl-a is similar over the NOW, with higher values over the central northern Baffin Bay as well as a reduction in extent in the vicinity of Smith Bay that is the result of ice cover in this region (Fig. 3b). In addition, Chl-a is also present along the entire extent of Nares Strait with maximum values along Kennedy Channel and northern Kane Basin in excess of 0.25 mg/m^3^.

The distribution of Chl-a along Nares Strait for the two arch categories (Fig. 11c) indicates the concentrations over the NOW tends to be larger for the no southern arch cases south of 77° N with similar concentrations for the two categories over the northern NOW. The year-to-year variability is similar for the two categories.

**Figure 12:**
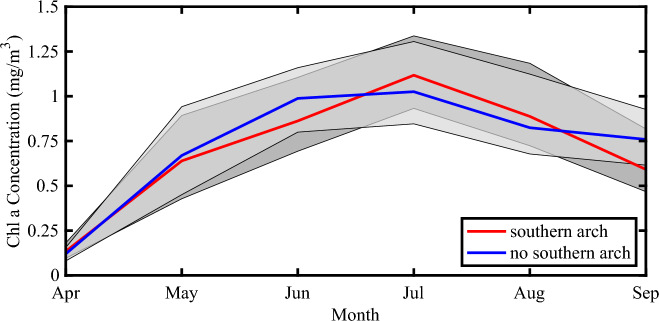
Evolution of ocean color along the NOW as a function of arch formation. The red line indicates the time series of the monthly mean median Chl-a concentration for years in which a southern arch formed with the dark shading representing the Chl-a concentration bounded by the first and third quartiles. The blue line indicates the time series of the monthly mean median Chl-a concentration for years in which no southern arch formed with the light shading representing the Chl-a concentration bounded by the first and third quartiles. Please refer to the “Methods” section for details on the categorization of the winters. All data based on the Aqua/Terra/Suomi NPP Chl-a data 2002–2022. The figure was generated using MATLAB R2022b.

Figure 12 shows the evolution of Chl-a over the NOW over the growing season for the two arch categories. The distributions in both categories are similar with peak values for the no southern arch case occurring in June while those for the southern arch case occurring in July. The inter-annual variability in the two categories is also similar.

## Discussion

Nares Strait is an important pathway for the export of multi-year sea ice out of the Arctic. Ice arches are a common occurrence at either the northern or southern end of the Strait during the winter^37^. The presence of either type of arch leads to a cessation of the export of Arctic sea ice down the Strait^8,13^. In addition, it has been argued that the presence of a southern arch, by restricting the southward movement of sea ice thereby allowing the northerly winds along the strait to sweep any remaining ice out of the region, is necessary for the existence of the NOW^14–17^. There is evidence that the stability of the arches is weakening^8^ and there is concern that this may impact the existence of the NOW as a unique recurring polynya with its high level productivity, its complex ecosystem and its use as a traditional food source for nearby Inuit communities^1,14^.

In this paper, we have extended a previous analysis of the IAF along Nares Strait covering the period 2016–2019^8^ to include the 3 additional winters of 2020, 2021 and 2022 (Figs. 2, 3). This extension identified one additional winter, 2022, during which no ice arches formed along Nares Strait. Previous work had identified 2007^1^ and 2019^8^ as winters where no ice arches formed along the strait. These three winters along with three previously identified winters in which a northern ice arch formed, the winters of 2008, 2009 and 2017^36,37^ provide 6 realizations of conditions along the Strait in the absence of a southern arch. We used these 6 winters to provide insight into what the sea ice along the Strait and over the NOW might look like if southern arches fail to form in the future.

We found that the presence or absence of a southern arch can have a profound impact on the sea ice distribution along Nares Strait. In the absence of a southern arch, the ice remains mobile throughout the winter allowing the strong winds that blow along Nares Strait to advect the ice southwards resulting in areas with sea ice concentrations as low as 25% (Figs. 4, 5, 6, 7). This is particularly true along Kennedy Channel. Over the Kane Basin, to the south of Kennedy Channel, sea ice concentrations rebound to values close to those attained in the southern arch case.

There are some differences in the distribution of sea ice along the Strait between the situation that occurs when a northern arch is present as compared that when no arches are present (Fig. 8). In particular, sea ice concentrations along the Kane Basin and north of the Kennedy Channel tend to be higher when no arch is present. This can be understood as the result of the differences in ice type between these two situations. In the absence of an arch along Nares Strait, thick multi-year ice continues to flow southwards during the winter. In contrast, the presence of a northern ice arch restricts this transport and any sea ice that is present along the Strait is thin first year ice that has just recently formed. For a given wind forcing, the thinner ice is more mobile^44^ and hence is easier to be advected downstream resulting in lower sea ice concentrations for the case of a northern arch.

Over the NOW, the situation is more nuanced. There is evidence of an increase in sea ice concentration along the Ellesmere Island coast of northern Baffin Bay in those winters when no southern arch is present (Fig. 5). Given the thinness of the ice (Fig. 9), this is most likely a signature of the transport of sea ice along Nares Strait that occurs during these winters along with the ocean currents in northern Baffin Bay that restrict this transport to the western side of northern Baffin Bay^21^. Wind-driven upwelling of warm Atlantic origin water as well as brine rejection driven convection that occurs on the eastern side of the Bay also contributes to the presence of open water even in the absence of an ice arch^21^. Although there is considerable variability, sea ice concentrations over the NOW tend to be ~ 10% higher for the no arch case as compared to the southern or northern arch cases (Fig. 8b).

The thickness distributions along the Strait are quite different in the presence or absence of a southern arch (Fig. 9). For the case of a southern arch (Fig. 9a), ice with thicknesses greater than 1.25 m is present north of Smith Sound. In the absence of a southern arch, ice with this thickness is only present north of the Kennedy Channel (Fig. 9b). There appears to be no difference in the thicknesses north of the Kane Basin for the case of a northern arch or in the absence of an arch with significantly thicker ice in the southern arch case (Fig. 9c). As discussed above during a winter with no arch present, thick multi-year ice would continue to be advected along the Strait; while during a northern arch winter, only first year ice would be advected. Although the number of cases are small, it appears that the strength of the winds along the Kennedy Channel are sufficiently strong to remove ice, regardless of thickness, from the region.

As one moves southwards along Kane Basin, ice thickness in the no southern arch category increases rapidly (Fig. 9c). This is likely the result of convergence as the mobile ice is influenced by the narrowing of the Strait in the vicinity of Smith Sound. This dynamical thickening of the mobile ice would also occur prior to the formation of a southern arch and may contribute to its formation^9^. It is likely that convergence also occurs as sea ice moves into the Robeson Channel from the Lincoln Sea would contribute to the formation of a northern arch.

Wind speeds along the Kennedy Channel and the northern Kane Basin tend to be higher during those winters with no southern arch present (Fig. 10). This is consistent with both historical^45^ and recent^43^ expeditions to the region. Recall that during these winters, the ice concentration is lower in this region and it is likely that the acceleration of the winds is the result of reduction is surface friction^46^. Such an acceleration of the wind would also provide a positive feedback that would tend to reduce ice concentrations. It should be noted that the CARRA assumes a uniform sea ice thickness of 0.75 m and therefore may not fully represent changes in surface friction associated with the presence of multi-year sea ice along the Strait^47^. It must also be stressed that arches typically form in the early winter and so this study does not address the role that winds play on the formation of ice arches along the Strait.

With respect to primary productivity, there appears to be no difference in the magnitude of Chl-a over the NOW for years with or without a southern ice arch (Figs. 11, 12). There is however a northward expansion of Chl-a in winters without a southern arch.

The results presented here suggest that reduced ice concentrations, as low as 25%, occur along the Kennedy Channel in the absence of a southern ice arch. Over the NOW, the changes are more nuanced with reduced ice cover present for both categories but with ice concentrations in the no southern arch category higher by 10–20%. This suggests that contrary to previous work^14–17^, a southern arch is not necessary for the existence of the NOW. Given the observed reduction in the stability and duration of the Nares Strait ice arches^8,15,37^, the absence of a southern arch case may become the status quo moving forward. Even in the absence of a southern ice arch, strong winds are still present to the vicinity of Smith Sound (Fig. 10) that would continue to advect ice southwards. In the current climate, this work suggests that primary productivity over the NOW is also independent of the presence of absence of a southern ice arch. There are however environmental changes associated with a warming climate that may nevertheless result in changes to the primary productivity and the health of the ecosystem moving forwards^14,27–29^. These results suggest that a re-examination of the fate of the NOW in a warming climate needs to be undertaken.

## Methods

### Ice area flux

As was the case in previous work^1,8^, the ice area flux (IAF) along Nares Strait was calculated using a flux gate situated within the southern Lincoln Sea (Fig. 1). Sequential pairs of Sentinel-1 SAR images along with a feature tracking algorithm^31^ were used to establish the ice motion in the vicinity of the flux gate. The ice motion along the flux gate was obtained by interpolating the ice motion at a 5 km horizontal resolution. The ice area flux was obtained by integrating the product of the ice motion and the sea ice concentration from the closest Canadian Ice Service ice chart^48^. Error estimates for the daily ice area flux are on the order of ± 10 km^2^/day^8^.

### Sea ice concentration

Passive microwave sea ice concentration data using the AMSR-E/2 instruments and the ARTIST retrieval^49^ were used. The availability of the high frequency 89 GHz channel on these instruments provides sea ice concentration at a spatial resolution of 3.125 km^49^ that is crucial to resolve the spatial variability in this field along the Strait^8^. The data is available, with some interruptions, from 2003 to 2022. Error estimates range from ~  ± 15% for sea ice concentrations close to 0% to ~  ± 6% for sea ice concentrations close to 100%^49^.

### Ice arch dates

The IAF flux data in Fig. 1 along with results from previous studies^1,37^ were used to classify the winters for which the AMSR-E/2 sea ice data is available (2003–2022) as to the presence or absence of an arch. Table 1 provides a categorization of these winters.

**Table 1:** Categorization of winters as to the presence or absence of an ice arch along Nares Strait.

Winter	Category	Winter	Category
2003	Southern Arch	2013	Southern Arch
2004	Southern Arch	2014	Southern Arch
2005	Southern Arch	2015	Southern Arch
2006	Southern Arch	2016	Southern Arch
2007	No Arch	2017	Northern Arch
2008	Southern Arch	2018	Southern Arch
2009	Northern Arch	2019	No Arch
2010	Northern Arch	2020	Southern Arch
2011	Southern Arch	2021	Southern Arch
2012	Southern Arch	2022	No Arch

### Sea ice thickness

Sea ice thickness data from the merged CryoSat-2/SMOS^40^ were used. The primary payload on the CryoSat-2 satellite^50^ is a radar altimeter that retrieves sea ice thickness through measurements of sea ice freeboard with a spatial footprint of ~ 0.3 km by 1.7 km^51,52^. The primary payload of the Soil Moisture and Ocean Salinity (SMOS) satellite is a passive microwave radiometer operating at 1.4 GHz^53^ that is able to retrieve sea ice thickness up to ~ 1 m at a spatial resolution of 12.5 km^54^. CryoSat-2 retrievals of thin ice have a greater uncertainty as compared to thick ice; while SMOS exhibits greater sensitivity to retrievals of thin ice^55^. As a result, the merging of the two products results in an improved retrieval of ice thickness with lower rms errors and higher correlation as compared to in-situ observations^55^. The data is available where the sea ice concentration is non-zero on a weekly basis, updated daily, at a spatial resolution of 25 km during the cool season (October–April) from 2010/2011 to 2021/2022^40^.

The merged CryoSat-2/SMOS dataset provides an estimate of the uncertainty in the thickness retrieval^40^. Supplementary Figure S1 shows the climatological sea ice thickness during May as well as the uncertainty and percent uncertainty. The climatological sea ice thickness during May (SF1a) is less than 1 m over the NOW increasing to 1.4 m over the Kane Basin and over 2 m over the Lincoln Sea. This distribution reflects the impact of a southern ice arch that is the most common situation (Table 1). The uncertainty during May (SF1b) varies from less than 0.3 m over the NOW to over 0.5 m over the Lincoln Sea. This is consistent with the design parameters for the dataset^40^. As a result, the percent uncertainty during May (SF1c) is largest over the thin ice of the NOW, in excess of 60%, while it is less than 30% along Nares Strait.

### Surface winds

There is evidence that the narrowness of the Strait combined with its steep topography requires high spatial resolution to represent the local wind climate^18,41,56^. As a result, we use the 10 m meridional wind from the western domain, that includes Nares Strait and northern Baffin Bay, of the Copernicus Arctic Regional Reanalysis (CARRA). It is available on a Lambert Conformal grid with a horizontal resolution of 2.5 km and a temporal resolution of 3 h^57^. The CARRA is based on the mesoscale Numerical Weather Prediction (NWP) system known as HARMONIE-AROME^58^ with some modifications and extensions that are described in Yang^57^. Boundary conditions for CARRA are provided by the ERA5 reanalysis^59^. We used data for the period of overlap with the AMSR-E/2 dataset, 2002–2022. Sea ice in the CARRA is assumed to a uniform thickness of 0.75 m^60^. Within the Nares Strait region, a comparison with in-situ wind data suggests an uncertainty of ± 2.5 m/s^42^.

### Ocean color data

Space-based ocean color data provides a remotely sensed estimate of primary productivity^61^. Ocean color remote sensing has proven to be an important tool for monitoring oceanic primary productivity as it provides high spatial and temporal resolution information on biochemical proxies, such as chlorophyll-a (Chl-a), in remote regions with harsh climates^62^. As with any remotely sensed products, there are challenges in using the data. The presence of clouds can limit the availability of data^63^, as does sea ice cover that also introduces complications in the retrival of estimates of Chl-a. Despite these complexities, Chl-a data has been used in a number of studies to assist in the characterization of the NOW’s primary productivity^14,23,27,28,64–66^. Here we use monthly mean retrievals of Chl-a from the MODIS instruments on the Terra and AQUA satellites as well as the VIIRS instrument on the Suomi NPP satellite for the period 2002–2022 as processed by Ocean Biology Processing Group at NASA’s Goddard Space Flight Center (https://oceancolor.gsfc.nasa.gov).

### Latitudinal averaging

To provide information on the variability in the various datasets along Nares Strait, we used a latitudinal averaging process. The centerline along Nares Strait was determined from the GEBCO digital elevation model (Fig. 1). For fixed latitudes along the centerline, data from longitudes across the strait were averaged together to provide a representative value for that latitude. The spacings in both latitude and longitude were selected to preserve the resolution of the underlying dataset.

## Supplementary Information

Supplementary Figure S1.

## Data Availability

Sentinel-1 SAR imagery is available from the Copernicus Open Access Hub at: http://scihub.copernicus.eu. AMSRE/2 sea ice concentration data is available from the University of Bremen at: https://seaice.uni-bremen.de/. The CyroSat-2/SMOS sea ice thickness data is available at: https://data.seaiceportal.de/data/cs2smos_awi/v205/. The CARRA data is available at: https://cds.climate.copernicus.eu/cdsapp#!/dataset/reanalysis-carra-single-levels. The Chl-a data is available at: https://oceandata.sci.gsfc.nasa.gov.
