# Supplementary material for: Evolving relationship of Nares Strait ice arches on sea ice along the Strait and the North Water, the Arctic’s most productive polynya
Source: Sci Rep. 2023 Jun 17;13:9809. doi: 10.1038/s41598-023-36179-0 (PMC10276818; doi:10.1038/s41598-023-36179-0)
Supplement: Supplementary file 1 — Supplementary Figure S1. [file 41598_2023_36179_MOESM1_ESM.pdf]

Supplementary Material for

Evolving relationship of Nares Strait ice arches on sea ice along the Strait and the North Water, the Arctic's most productive polynya

G.W.K. Moore *et al.*
.

*Corresponding author. Email: gwk.moore@utoronto.ca

This PDF file includes:

Fig. S1

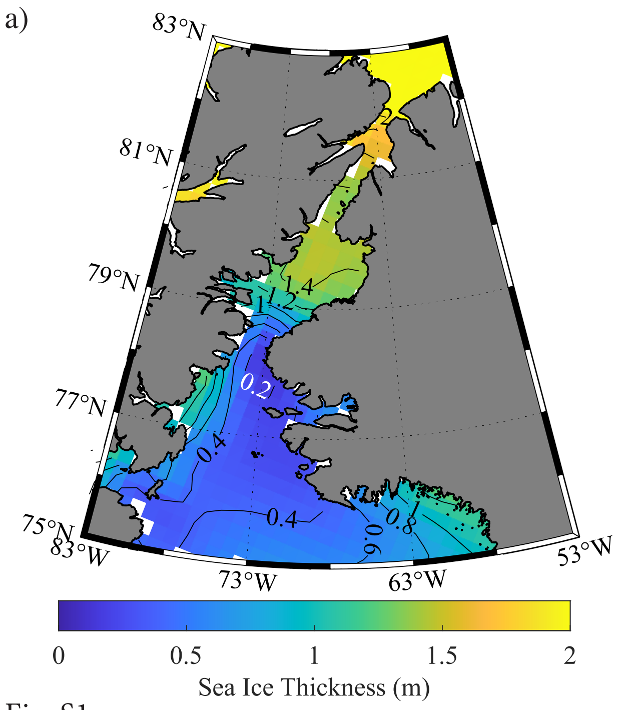

Fig. S1.
Uncertainty in the sea ice thickness field along Nares Strait. a) The climatogical mean sea ice thickness (m) during May 2010-2022. b)The uncertainty in the sea ice thickness (m) during May 2010-2022. c) The percent uncertainty (%) during May. The figure was generated using MATLAB R2022b.
